# Theoretical Study of Electric, Dielectric, and Optical Properties in Ion Doped Multiferroic SrFe_12_O_19_ Nanoparticles

**DOI:** 10.3390/ma17071544

**Published:** 2024-03-28

**Authors:** Angel T. Apostolov, Iliana N. Apostolova, Julia Mihailowa Wesselinowa

**Affiliations:** 1 University of Architecture, Civil Engineering and Geodesy, Hr. Smirnenski Blvd. 1, 1046 Sofia, Bulgaria; angelapos@abv.bg; 2 University of Forestry, Kl. Ohridsky Blvd. 10, 1756 Sofia, Bulgaria; inaapos@abv.bg; 3 Faculty of Physics, Sofia University “St. Kliment Ohridski”, J. Bouchier Blvd. 5, 1164 Sofia, Bulgaria

**Keywords:** La- and Ni-doped SrFe_12_O_19_, polarization, dielectric constant, band gap, microscopic model

## Abstract

Electric, dielectric, and optical (band gap) properties of pure multiferroic as well as La- and Ni-doped ${\mathrm{SrFe}}_{12}$$O_{19}$ (SFO) (at different sites) are investigated using a microscopic model and Green’s function technique. The concentration dependence of the polarization *P* is considered for substitution of rare earths ions on the Sr sites. For a small La ion doping concentration, *x* = 0.1, La-doped SFO is ferroelectric, whereas for a larger doping concentration, for example *x* = 0.5, it is antiferroelectric. The real part of the dielectric constant ϵ increases with an increasing magnetic field *h*. ϵ decreases with an increasing frequency and La dopants. Therefore, La-doped SFO is suitable for microwave application with a low dielectric constant. The magnetic properties of pure SFO NPs are also studied. Ni doping at the Fe site of SFO leads to enhanced ferroelectric polarization and dielectric constant. The band gap decreases or increases by substitution of Ni or In ions on the Fe site, respectively. The results reveal that the tuned band gap of Ni-doped SFO makes it a crucial candidate for optoelectronic and solid oxide fuel cell applications.

## 1. Introduction

M-type hexaferrites, including materials like strontium ferrite (SrFe_12_O_19_ or SFO), exhibit fascinating physical properties that render them highly versatile in various technological applications. These properties include a notably high Curie temperature, exceptional electrical resistivity, significant magnetization, and magnetocrystalline anisotropy. Consequently, they find utility in diverse fields such as high-density magnetic recording media, magneto-optical devices, and microwave technologies [1,2,3].

The manipulation of M-type hexaferrite properties, particularly those of SFO, can be achieved through various means, primarily via substitution at ${\mathrm{Fe}}^{3+}$ or Sr^2+^ sites. Such substitutions induce distinct modifications in the magnetic and electric characteristics of SFO owing to the resultant strain effects. Numerous studies have investigated the magnetic properties of both pure and ion doped SFO, whether in bulk form or as nanoparticles (NPs) [4,5,6,7,8,9,10,11,12,13].

Introducing a dopant, such as La, serves to augment several key parameters. For instance, La substitutions have been shown to enhance the saturation magnetization, the magnetocrystalline anisotropy, and the Curie temperature [14,15,16]. Additionally, the controlled doping of these materials can lead to tailored band gaps and enhanced conductivity, further expanding their potential applications in optoelectronics and solid oxide fuel cells [17,18,19,20]. Hence, ion-doped M-type hexaferrites, particularly when doped or modified, stand out as pivotal candidates for a broad spectrum of technological advancements.

Significant discoveries have unveiled the remarkable coexistence of large ferroelectricity and robust ferromagnetism within SFO, with the ferroelectric critical temperature notably smaller than the ferromagnetic counterpart [21,22]. Moreover, investigations have revealed that ${\mathrm{BaFe}}_{12}$$O_{19}$ (BFO) and ${\mathrm{PbFe}}_{12}$$O_{19}$ (PFO) also exhibit ferroelectric (FE) properties. In Ni-doped SFO, enhancements in FE polarization, coercivity, and remnant polarizations have been observed [17]. However, in La-doped SFO at the Sr site, ${\mathrm{La}}_{x}$${\mathrm{Sr}}_{1-x}$${\mathrm{Fe}}_{12}$$O_{19}$, an intriguing antiferroelectric (AFE) behavior is reported for specific dopant concentrations *x* (for *x* = 0.7 by Huang et al. [23] and for *x* = 0.5 by Yin et al. [24]).

Recent studies by Duan et al. [25] have delved into La-doped SFO across various doping levels (*x* = 0–0.5), revealing a complex scenario. For instance, for *x* = 0.2, a hybrid ferroelectric/antiferroelectric (FE/AFE) state has been identified (also observed in [22,26]), while for *x* = 0.5, a pure AFE behavior is observed. The substitution of ${\mathrm{La}}^{3+}$ for ${\mathrm{Sr}}^{2+}$ in SFO could change the shape of the hysteresis loops. It keeps the charge balance, but induces vacancies. Increase in the dopant leads to disruption of the translational periodicity of the lattice, to break the long-range FE interactions. Above a critical concentration of ${\mathrm{La}}^{3+}$ dopant, the doping effect thus turns the properties of SFO from FE to AFE phase. A similar composition-induced FE to AFE phase transition is reported for example in Sm-doped ${\mathrm{BiFeO}}_{3}$ systems [27].

It must be noted that multiferroic compounds exhibiting AFE behavior alongside magnetic properties are scarce, with examples including ${\mathrm{NaCu}}_{2}$$O_{2}$, ${\mathrm{LiFeP}}_{2}$$O_{7}$, and ${\mathrm{BiCrO}}_{3}$.

The dielectric constant ϵ of La-doped SFO has been thoroughly investigated in various studies [23,24,25,28], for example Ni-doped SFO [17,29], Co-doped SFO [30], Mn-doped SFO [31], and Ca-doped SFO NPs [32]. These investigations explore ϵ as a function of temperature, ion doping concentration, frequency, and magnetic field, shedding light on the intricate interplay between structural, magnetic, and dielectric properties in these materials.

The investigation of the band gap energy $E_{g}$ of pure SFO has been explored by Hou et al. [8] and Manikandan et al. [33], while the effects of Ni, In, and Mn doping on SFO have been studied by Irshad et al. [17], Zhang et al. [34] and Rasheed et al. [31], respectively. Notably, these studies shed light on the alterations in the optical band gap.

In particular, findings indicate that Ni substitution leads to a reduction in the optical band gap, a trend that aligns well with the observed decline in photoluminescence analysis [17]. This suggests a clear association between doping and the modification of optical properties, offering insights into the intricate interplay between dopant ions and the electronic structure of SFO. Such investigations are crucial for understanding the potential applications of doped SFO in optoelectronic devices and related fields.

It is noteworthy to mention that in our previous study [35], we investigated the multiferroic properties of both pure and ion-doped BFO, specifically incorporating Ni, Zr, and Sm dopants. To the best of our knowledge, there is a dearth of theoretical studies that have systematically examined the properties of ion-doped SFO, while there exist some investigations focusing on pure bulk SFO utilizing Density Functional Theory (DFT) techniques [8,36], comprehensive theoretical analyses regarding doped SFO remain scarce. Therefore, in the current manuscript, we employ for the first time a microscopic model combined with Green’s function theory to consider the electric, dielectric, and optical (band gap energy) properties of both pure and La- or Ni-doped SFO NPs. Their temperature, size, doping concentration, and magnetic field dependencies are considered. Due to the different strain the exchange interaction constants at the doping state are changed and can be larger or smaller than those in the undoped state. This leads to modification of the physical properties. Thus, we study the macroscopic properties on microscopic level. Moreover, the calculations within Green’s function method go beyond the Random Phase Approximation, taking into account the correlation functions and damping effects. It is crucial to highlight that while DFT primarily addresses ground state properties at zero temperature, our approach enables a finite temperature analysis of the excitation spectrum and all associated physical quantities. This methodology provides valuable insights into the behavior of doped SFO at realistic operating conditions, offering a comprehensive understanding of their potential applications in various technological domains.

## 2. The Model

The magnetic properties of Ni-doped SFO at the Fe sites are described by the Heisenberg model:

(1) $$\begin{array}{ccc} H_{m} & = & -\frac{1}{2}\sum_{ij}\left(1-x\right)J_{i,j}^{Fe-Fe}S_{i}^{Fe}\cdot S_{j}^{Fe}-\frac{1}{2}\sum_{ik}xJ_{i,k}^{Fe-Ni}S_{i}^{Fe}\cdot S_{k}^{Ni}\\ & - & K_{1}\sin^{2}\theta \sum_{i}S_{i}^{zFe}-D\sum_{i}{\left(S_{i}^{zFe}\right)}^{2}-g\mu_{B}h\cdot \sum_{i}S_{i}^{Fe}, \end{array}$$

where $S_{i}$ is the Heisenberg spin operator of the ${\mathrm{Fe}}^{3+}$ spin at the site *i*. $J_{ij}$ is the exchange interaction constant between Fe-Fe or Fe-Ni ions, *D* is the single-ion anisotropy, $K_{1}$ is the first anisotropy constant, and θ is the angle between the magnetization and the easy axis. *x* is the Ni-doping concentration at the Fe sites.

The origin of the polarization in SFO is due to the shift of ${\mathrm{Fe}}^{3+}$ ions from the center of the ${\mathrm{FeO}}_{6}$ octahedron [22,37]. Therefore, the ferroelectricity can be described by the transverse Ising model

(2) $$H_{f}=-\Omega \sum_{i}B_{i}^{x}-\frac{1}{2}\sum_{ij}\left(1-x^{\prime }\right)J_{ij}^{\prime }B_{i}^{z}B_{j}^{z}-\mu E\sum_{i}B_{i}^{z}.$$

The pseudo-spin operator $B_{i}^{z}$ characterizes the two position of the ferroelectric unit at the lattice point *i*. The exchange interaction is taken to be FE, $J_{ij}^{\prime }>0$. The dynamics of the model with strength Ω is determined by the operator $B^{x}$. $x^{\prime }$ is the La-doping concentration in ${\mathrm{Sr}}_{1-x^{\prime }}$${\mathrm{La}}_{x^{\prime }}$${\mathrm{Fe}}_{12}$$O_{19}$. *E* is an external electric field.

The magnetoelectric coupling is taken to be linear because the ferroelectric critical temperature is much smaller than the ferromagnetic one [21,22]:

(3) $$H_{mf}=-g\sum_{ikl}B_{i}^{z}S_{k}\cdot S_{l}.$$

The magnetization *M* for arbitrary spin *S* is given by:

(4) $$M=\langle S^{z}\rangle =\frac{1}{N}\sum_{i}\left[\left(S+0.5\right)\coth\left[\left(S+0.5\right)\beta E_{mi}\right]-0.5\coth\left(0.5\beta E_{mi}\right)\right],$$

where $E_{mi}$ is the spin wave energy, calculated from Green’s function:

(5) $$g_{ij}=\ll S_{i}^{+};S_{j}^{-}\gg .$$

The relative polarization *P* is given by

(6) $$P=\frac{1}{2N}\sum_{i}\tanh\frac{E_{fi}}{2k_{B}T}.$$

$E_{fi}$ is the pseudo-spin wave energy observed from the poles of Green’s function:

(7) $$G_{ij}=\ll B_{i}^{+};B_{j}^{-}\gg .$$

The equation for obtaining the dielectric function ϵ is [38]:

(8) $$\left({\left(\Lambda /\left(\epsilon \left(E\right)-1\right)\right)}_{\alpha \beta }+\Lambda \frac{k_{\alpha }k_{\beta }}{k^{2}}\right){\tilde{G}}^{\beta \gamma }\left(E\right)=\delta_{\alpha \gamma }.$$

$\Lambda =4\pi Z^{2}/v$, where *Z* is the electron charge and *v* is the volume. In order to obtain ϵ we must calculate the longitudinal anticommutator Green’s function ${\tilde{G}}^{zz}\left(E\right)=\langle \langle B_{i}^{z};B_{j}^{z}\rangle \rangle$.

## 3. Numerical Results and Discussion

For the numerical calculations are used the following model parameters: $J^{Fe-Fe}$ = 510 K, *D* = −2.88 K [39], $K_{1}$ = 3.3 × 10^6^ erg ${\mathrm{cm}}^{-3}$ at *T* = 300 K [40], $K_{1surface}$ = 1.8 × $10^{6}$ erg ${\mathrm{cm}}^{-3}$ [41], $J^{Fe-Ni}$ = 88.3 K, *g* = 21 K, $J^{\prime }$ = 565 K, Ω = 20 K, *S* = 5/2 for ${\mathrm{Fe}}^{3+}$, and *S* = 1/2 for the pseudo-spins.

Doping of SFO with La ions creates a compressive strain because the radius of the doping ${\mathrm{La}}^{3+}$ ion (1.17 $\dot{A}$) is smaller compared to that of the host ${\mathrm{Sr}}^{2+}$ ion (1.32 $\dot{A}$). Huang et al. [23] also reported that the cell parameters of La-doped SFO are smaller, the unit cell volume is contracted by 0.59% in comparison with that of undoped SFO. These means that the exchange spin interaction constant *J* is larger in the doped states $J_{d}$, than that in the undoped ones *J*, $J_{d}>J$, because *J* is inverse proportional to the distance between the spins to the lattice parameters. Moreover, there are oxygen vacancies in order to compensate for the charge difference.

SFO exhibits a magnetically hard behavior. The doped samples also show magnetically hard behavior. The kind of magnetism is not changed greatly by doping with diamagnetic ${\mathrm{La}}^{3+}$ ions. The magnetization *M* is depending on the La-doping concentration *x*. We obtain a slightly enhanced *M* with increasing *x*, in agreement with Liu et al. [42]. The substitution of ${\mathrm{Sr}}^{2+}$ with a smaller ${\mathrm{La}}^{3+}$ ion changes the position of the O ions and, therefore, also changes the Fe-O-Fe angle of the super-exchange interaction $J^{Fe-Fe}$ and leads to an increase in the magnetization *M*.

**Figure 1:**
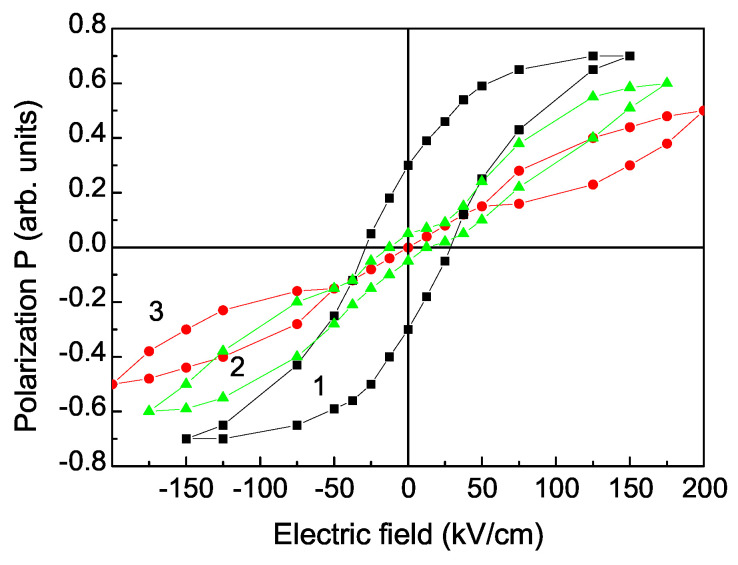
Electric field dependence of the polarization *P* for *T* = 300 K and different La doping concentrations *x*: (1) 0.1 (FE), (2) 0.18 (FE+AFE), and (3) 0.5 (AFE).

Firstly, we have calculated the electric polarization hysteresis loop for different La-doping concentrations *x*. The results are demonstrated in Figure 1. Curve 1 in Figure 1 shows that for small La-doping concentrations, for example *x* = 0.1, the system is in the FE state. We have a full saturation of the polarization *P*. With increasing the La dopants there is a transition from the FE to the AFE state (see Figure 1, curve 2), around ion doping concentration of *x* = 0.2, where the exchange pseudo-spin interaction constant $J^{\prime }$ changes the sign from positive to negative. The last sign is characteristic for the AFE state [43]. The curve 2 in Figure 1 shows this hybrid FE/AFE state.

For La-doping concentration *x* = 0.5 we observe a typical AFE hysteresis loop in SFO (Figure 1, curve 3). The double loops are separated by a linear AFE component indicating that the polarization vectors in the AFE region are antiparallel aligned and thus cancel each other. The occurrence of antiferroelectricity is due to the interruption of translational periodic symmetry on the substituted sites. The observed results are in agreement with the reported experimental data of Huang et al. [23], Yin et al. [24], Duan et al. [25], and Tan et al. [26]. Therefore, the FE state of La-doped SFO can be changed by varying the La-doping concentration *x* which leads to existing of ferromagnetism and antiferroelectricity. This makes it an interesting type of multiferroic material.

**Figure 2:**
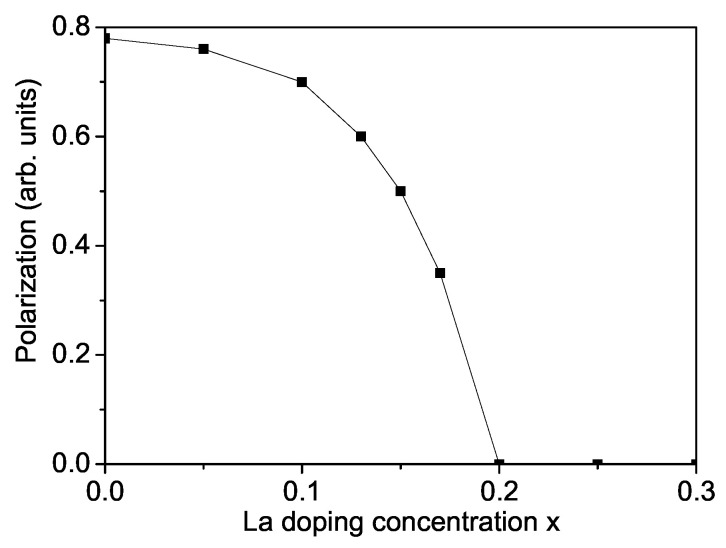
La-doping concentration dependence of the polarization *P* for SFO, *T* = 300 K.

In the next Figure 2 is presented the La-doping effect on the polarization *P*. It can be seen that *P* decreases with increasing La dopants. For *x* = 0.2 *P* reaches zero, i.e., for x≥0.2 it is AFE. Tan et al. [22] reported from temperature-dependent dielectric studies in pure SFO that peaks appear at 447 K and 641 K. The first peak at $T_{d}$ is assigned to the FE to AFE phase transition, while the second one at $T_{m}$ to the AFE to paraelectric phase transition. By La doping, $T_{d}$ is shifting to smaller values and the region with AFE polarization is enhanced. For large *x* values La doped SFO is AFE.

There are not so many multiferroic compounds that exhibit magnetic and AFE properties. For example, the M-type hexaferrite BFO shows frustrated antiferroelectricity associated with its trigonal bipyramidal ${\mathrm{Fe}}^{3+}$ sites [44]. Let us emphasize that the long-range interactions in SFO are influenced by the substitution of La ions and so lead to suppression of the FE order and stabilization of the AFT phase. Similarly, La doping of PZT-based ceramics has been investigated for enhancing the stability of antiferroelectricity for energy-storage applications [36].

**Figure 3:**
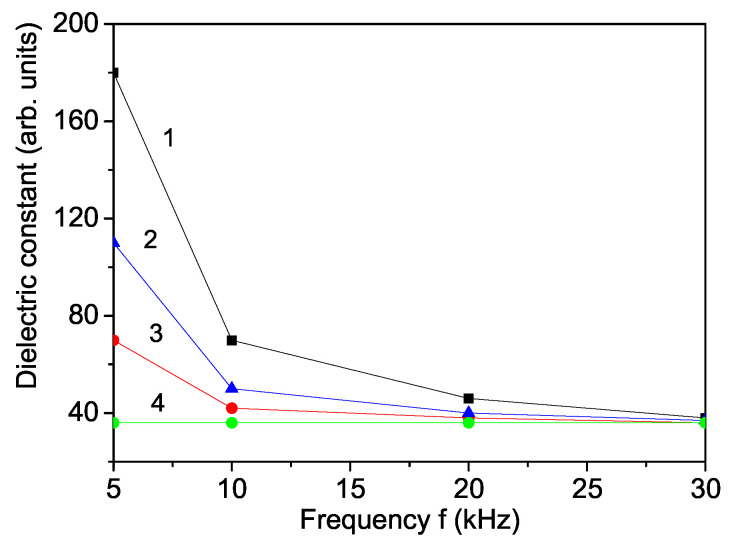
Frequency dependence of the dielectric constant ϵ for La-doped SFO, *T* = 300 K and different La-doping concentrations *x*: (1) 0; (2) 0.1; (3) 0.18; (4) 0.5.

We will now consider the effect of La doping on the real part of the dielectric constant ϵ of SFO, calculated from Equation (7). The dielectric constant ϵ as a function of frequency *f* is shown in Figure 3. It decreases with increasing the frequency *f* for both pure and La-doped SFO. As the frequency of the electric field is increased, the charge carriers do not align with the applied field. Thus, the polarization could not be saturated, does not follow the fluctuations of the applied electric field, and ϵ is reduced. A similar behavior of ϵ(f) is reported by Huang et al. [23] for pure SFO. The large dielectric constant at low frequency obtained for La-doped SFO makes it an interesting material for high-technical applications that require such types of behavior.

Moreover, we have calculated the real part of the dielectric constant ϵ as a function of the La dopants. ${\mathrm{La}}^{3+}$ ion doping changes the electron hopping between cations of the same element, which changes the dielectric properties. The dielectric constant ϵ decreases with increasing the La doping concentration *x* (see Figure 3, curves 2 and 3) due to the different ionic radii between the La and Sr ions. This behavior is in good qualitative agreement with the experimental data of Azim et al. [28] (*x* = 0–0.2). A similar decrease in ϵ we also observe in Ni-doped SFO. Therefore, La- and Ni-doped SFO is suitable for microwave application with low dielectric constant. Sharma et al. [45] reported that the dielectric constant of Ni in BFO decreases as the doping concentration increases due to increase in the band gap.

**Figure 4:**
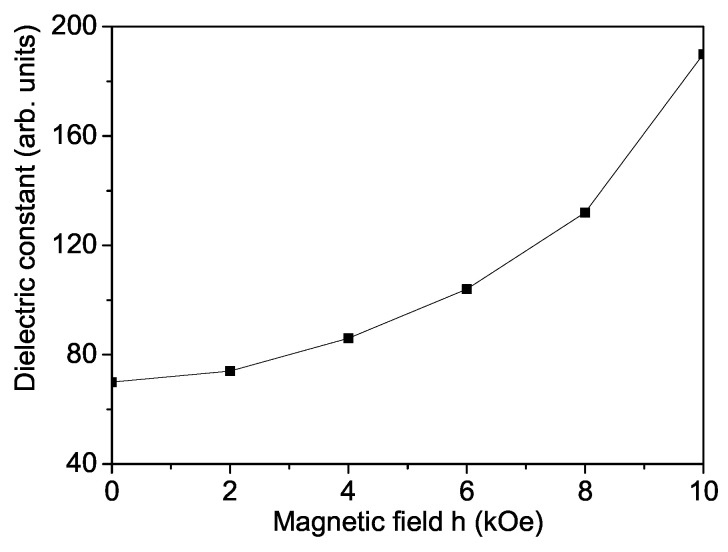
Magnetic field dependence of the dielectric constant ϵ for La-doped SFO, *T* = 300 K, *x* = 0.1.

In order to show the magnetodielectric behavior of La-doped SFO, we have studied the magnetic field dependence of the dielectric constant ϵ. From Figure 4 it can be seen that ϵ increases with increasing magnetic field *h*. The stronger the magnetic field, the larger the dielectric constant. This behavior of ϵ(h) is also observed in [23,24,25].

Next, we will consider the properties of a pure SFO NP with ferromagnetic to paramagnetic transition temperature $T_{Cfm}$ = 737 K [6]. The center of the NP is fixed at a certain Fe spin and all spins are included into shells numbered by *n* (n=N is the surface shell, n=1 is the central spin). The NP has in our case an icosahedral symmetry [46]. The distance between the shells is taken to be nearly 10 $\dot{A}$. Due to surface effects, the exchange interaction constant $J_{ij}\equiv J\left(r_{i}-r_{j}\right)$ depends on the inverse proportional on the lattice parameters. The exchange interaction constant of the surface layer $J_{s}$ is different from the bulk one $J_{b}$.

**Figure 5:**
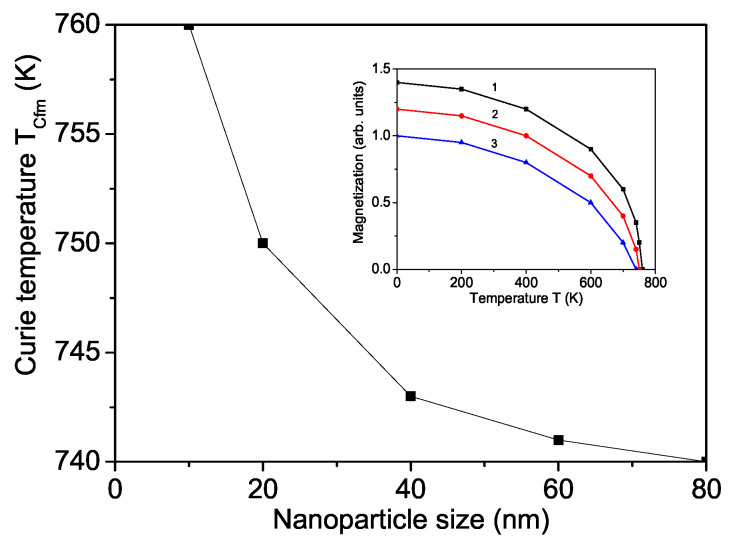
Nanoparticle size dependence of the magnetic Curie temperature $T_{Cfm}$ for SFO, $J_{s}^{Fe-Fe}=1.2J_{b}^{Fe-Fe}$. Inset: Temperature dependence of the magnetization for a SFO NP with different *d* values: (3) 80; (2) 20; (1) 10 nm.

Figure 5 shows the size dependence of the Curie temperature for a pure SFO calculated for the relation $J_{s}>J_{b}$. It can be seen that $T_{Cfm}$ increases with decreasing NP size *d*. A similar increase in the Curie temperature in SFO NPs is observed by Gajbhiye and Vijayalakshmi [11]. The magnetization also increases with decreasing *d* (see inset in Figure 5). The magnetic properties of SFO are improved with increasing NP sizes [47]. Liu et al. [10] reported an enhanced remanent magnetization $M_{r}$ and coercive field $H_{c}$ in SFO NPs. Therefore, the magnetic properties of M-type SFO can be improved by tuning the crystallite size.

**Figure 6:**
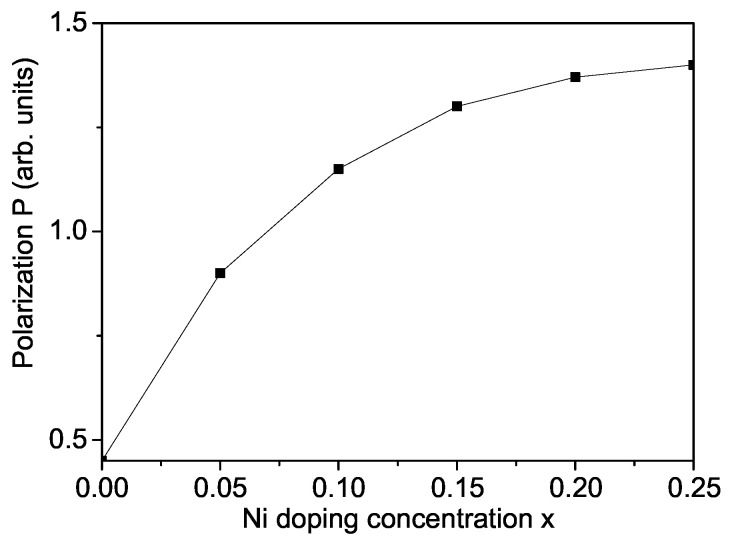
Ni-doping concentration dependence of the polarization *P* of a SFO NP, *d* = 20 nm, and *T* = 300 K.

Let us emphasize that, recently, ${\mathrm{Co}}^{2+}$-, ${\mathrm{Ni}}^{2+}$-, or ${\mathrm{Mn}}^{3+}$-doped SFO NPs at the Fe site are also studied [12,13,17,31,48,49]. An increase in the magnetization is reported by Liu et al. [48] and Thanh et al. [49] and of the Curie temperature $T_{C}$ by Ruikar et al. [12] with increasing Co dopants. Irshad et al. [17] observed that the FE polarization *P* is increased and the optical band gap $E_{g}$ is reduced by Ni doping. Rasheed et al. [31] obtained an increase in *P* and a decrease in $E_{g}$ and dielectric constant by increasing Mn content. The ion substitution at the Fe site in SFO can also be explained within our model. For example, we will consider the case of ${\mathrm{Ni}}^{2+}$-ion-doped SFO, ${\mathrm{SrNi}}_{x}$${\mathrm{Fe}}_{12-x}$$O_{19}$. A distortion of the crystalline lattice appears due to differences in the ionic radius of the ${\mathrm{Ni}}^{2+}$ ions (0.69 $\dot{A}$) and the host ${\mathrm{Fe}}^{3+}$ ions (0.64 $\dot{A}$) [17] which causes a variation in Fe-O and Ni-O bond lengths. Moreover, by doping with Ni ions, anionic (oxygen) vacancies occur (to ensure the charge neutrality) and two valences of the Fe ions also occur—${\mathrm{Fe}}^{2+}$ and ${\mathrm{Fe}}^{3+}$. So, although the radius of the doping ion is greater than that of the host one, the volume of the elementary cell decreases and we have to use the relation $J_{d}>J$, $J_{d}^{\prime }>J^{\prime }$. This leads to an enhanced magnetization *M* (not shown here) and polarization *P* (see Figure 6), in agreement with the experimental data of Irshad et al. [17]. Let us emphasize that the polarization is ferroelectric for all doping concentrations *x* contrary to the La doped SFO case. The dielectric constant also increases with increasing Ni dopants.

It should be mentioned that within our model and approximations we would also observe enhancement of the magnetization *M* and magnetic phase transition temperature $T_{C}$ by ${\mathrm{Co}}^{2+}$ ion doping and their decrease by ${\mathrm{Mn}}^{3+}$ doping as reported experimentally by [12,48,49,50], respectively.

For the calculation of the band gap $E_{g}$ we use the s-d model, where the s-d coupling term $H_{m-el}$ reads:

(9) $$H_{m-el}=\sum_{i}I_{i}S_{i}s_{i},$$

*I* is the s-d interaction constant and $s_{i}$ are the spin operators of the conduction electrons at site *i* which can be expressed as $s_{i}^{+}=c_{i+}^{+}c_{i-}$, $s_{i}^{z}=\left(c_{i+}^{+}c_{i+}-c_{i-}^{+}c_{i-}\right)/2$. The band gap energy $E_{g}$ is observed from the difference between the valence and conduction bands. The electronic energies are:

(10) $$\omega_{ij\sigma }^{\pm }=\epsilon_{ij\sigma }-\frac{\sigma }{2}I\langle S^{z}\rangle ,$$

where σ=±1, $\epsilon_{ij\sigma }$ is the conduction band energy in the paramagnetic state, and $\langle S^{z}\rangle$ is the magnetization.

**Figure 7:**
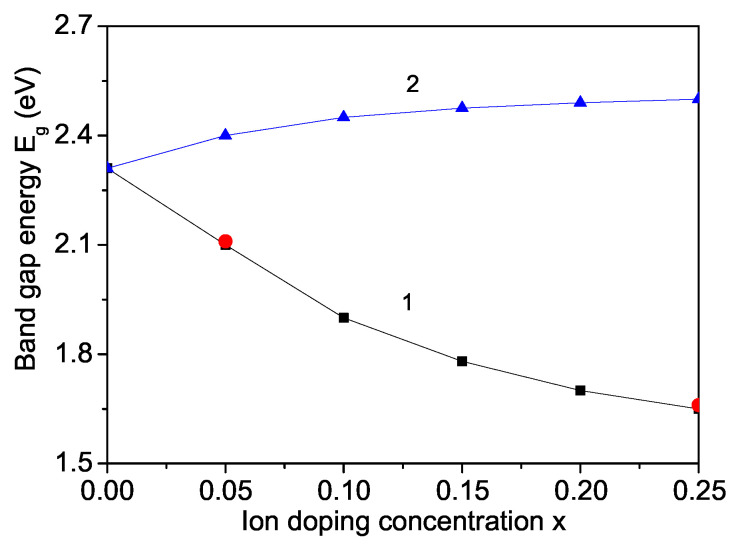
Ion-doping concentration dependence of the band gap energy $E_{g}$ for a SFO NP, *d* = 20 nm, *T* = 300 K, and different doping ions: (1) La and (2) In. The red points on curve 1 are the experimental data from Ref. [17].

Generally, the band gap energies of ferrites are reported to be about 2 eV [51]. It should be noted that there are some discrepancies between the $E_{g}$ values of bulk SFO (1.44 eV (theor.) [8], 1.75 eV [34], and 2 eV [52]) and of SFO NPs (1.89 eV [33] and 2.31 eV [17]). The band gap for undoped SFO NPs is taken to be 2.31 eV [17]. We have found a narrowing of $E_{g}$ in a Ni-doped SFO NP (see Figure 7, curve 1). A similar behavior is reported by Irshad et al. [17]. Moreover, there is a good quantitative agreement between our results and those of Ref. [17]. We observe $E_{g}$ = 2.1 and 1.65 for *x* = 0.05 and 0.25, whereas the experimental data from Irshad et al. [17] are 2.11 and 1.66, respectively. This is evidence for the appropriate model and the approximations we have made. The results show that tuned band gap of Ni-doped SFO makes it a crucial candidate for optoelectronic and solid oxide fuel cell applications, because decreasing of the band gap leads to enhancing the conductivity of the material [17]. Energy band gap tuning of BFO doped with Cr and Zn ions to enhance the optical, dielectric, ferroelectric, and photocatalytic properties is observed by Nazeer et al. [18]. Doping of alkaline earth metal at the A side in *R*${\mathrm{NbO}}_{3}$ (*R* is a rare-earth element) also decreases the band gap energy and enhances the conductivity of the material, which makes it appropriate for potential applications in energy storage and photovoltaics [19].

Let us emphasize that for other doping ions, for example for the non-magnetic ${\mathrm{In}}^{3+}$ with an ionic radius larger than that of Fe, there is a tensile strain and we find that the band gap energy $E_{g}$ increases slightly with increasing the In dopant (see Figure 7, curve 2). This is confirmed by the experimental data of Zhang et al. [34].

## 4. Conclusions

In conclusion, we have investigated theoretically for the first time pure and La-doped bulk SFO at the Sr site. For small a La-ion-doping concentration, *x* = 0.1, SFO is FE, whereas for larger doping concentration, *x* = 0.5, it is AFE. The transition from FE to AFE states appears around *x* = 0.2. The dielectric constant ϵ decreases with increasing frequency and La dopants for *x* = 0.1. Therefore, La-doped SFO is suitable for microwave application with low dielectric constant. There is a strong magnetodielectric coupling. ϵ increases with increasing *h*. The Curie temperature $T_{Cfm}$ and the magnetization *M* of pure SFO increase with decreasing NP size *d*. Ni doping at the Fe site of SFO leads to enhanced ferroelectric polarization and dielectric constant, as well as to a reduced band gap energy. $E_{g}$ increases with substitution of In ions at the Fe site. The results are compared with the existing experimental data.

It could be noted that the co-doping of rare earth ions at the Sr site and transition metal ions at the Fe site in SFO will be investigated in a future paper.

## Data Availability

Data are contained within the article.
